# A New Flexible Soy-Based Adhesive Enhanced with Neopentyl Glycol Diglycidyl Ether: Properties and Application

**DOI:** 10.3390/polym8090346

**Published:** 2016-09-21

**Authors:** Jing Luo, Jianlin Luo, Jizhi Zhang, Yuanyuan Bai, Qiang Gao, Jianzhang Li, Li Li

**Affiliations:** 1 Ministry of Education Key Laboratory of Wooden Material Science and Application, Beijing Key Laboratory of Wood Science and Engineering, College of Materials Science and Technology, Beijing Forestry University, Beijing 100083, China; luojing.rowe@gmail.com (J.L.); bjfuljl@gmail.com (J.L.); yuanhai_9@126.com (Y.B.); 2 School of Materials Science and Engineering, Shandong University, Jinan 250061, China; zjzvip@sdu.edu.cn

**Keywords:** soy-based adhesive, neopentyl glycol diglycidyl ether, water resistance, toughness, plywood

## Abstract

Soy-based adhesives inherently possess low water resistance and brittleness, which limit their application on plywood fabrication. This investigation involves using a long chain cross-linker, neopentyl glycol diglycidyl ether (NGDE), to produce an intrinsic toughening effect to reduce the brittleness and improve the water resistance of a soybean meal–based adhesive. The solids content, viscosity, functional groups, fracture surface micrographs, and thermal stability of the adhesives were measured. Three-layer plywood was fabricated using the resultant adhesive, and the tensile shear strength of the plywood was measured. All adhesive properties were compared with a soybean meal/polyamidoamine-epichlorohydrin (PAE) adhesive and commercial melamine urea formaldehyde resin. The results showed that adding 6 g NGDE improved the water resistance of the soybean meal-based adhesive by 12.5%. This improvement is attributed to the following reasons: (1) a dense cross-linked network is formed by the chemical reaction between NGDE and protein molecules; (2) the toughness of the adhesive increases and a smooth and homogeneous fracture surface is created, which effectively prevents moisture intrusion; (3) the addition of NGDE increases the thermostability of the cured adhesive. The tensile shear strength of the plywood bonded with the soybean meal-based adhesive with 6 g NGDE was 286.2% higher than that without NGDE and attained 1.12 MPa, which was attributed to the reduction in the adhesive’s viscosity, and the improvement in the water resistance and toughness of the adhesive. The tensile shear strength of the plywood bonded with 6 g NGDE was 19.1% higher than that with 6 g PAE and was similar to the MUF resin, which validated the novel adhesive being suitable for use as an industrial plywood adhesive.

## 1. Introduction

Formaldehyde-based resins play a dominant role in the present plywood fabrication industry because of their advantages, such as good water resistance, fast curing, and good age resistance. However, the formaldehyde emission issue and over-reliance on fossil resources of these adhesives have created a need to develop adhesives based on environmentally friendly, renewable resources [1,2,3,4].

As a renewable product, soy protein has the advantages of being abundant, inexpensive, and environmentally friendly, so it offers great potential as a raw material for wood adhesives. Using protein as a raw material to develop a wood adhesive can be traced back to the 19th century [5]. However, the low bond strength and water resistance of the soy protein–based adhesives limit their application and they have gradually been replaced by formaldehyde-based adhesives. In recent years, utilizing renewable biomass as a raw material to develop industrial products has become popular to reduce the consumption of petro-chemical feedstock and to diminish environmental pollution [6,7]. Therefore, the soy protein–based adhesives have become a focus of research. The efforts to improve the water resistance of the soy protein–based adhesives can be classified into four categories: denaturing agent modification, protein molecular modification, reactive cross-linker and synthetic resin modification. Denaturants, such as urea, sodium dodecyl sulfate, and alkali, can unfold the protein molecule and in some cases create a more crystalline domain to prevent water intrusion, which improves the water resistance of the adhesive [8,9,10,11,12]. However, the resultant plywood bonded with the soy protein–based adhesive containing denaturants cannot meet interior use requirements. Soy protein molecular modification focuses on grafting highly active groups onto the soy protein molecules. These groups react with each other or the active groups in the protein to form a cross-linked network in the resultant adhesive after curing [13,14,15]. This process improves the water resistance of the adhesive, but it is complex and costly, making it impractical for plywood fabrication. Active cross-linkers and synthetic resin modification include the use of glycidyl methacrylate, polyethylene glycol diacrylate, latex-based adhesive, formaldehyde-based resin, epoxy resin, etc. [16,17,18,19,20,21]. Based on our previous research [22], both chemical compounds with epoxy groups (such as ethylene glycol diglycidyl ether) and polyamidoamine-epichlorohydrin (PAE) were proved to be the most effective and practical cross-linkers for improving the water resistance of the soy protein–based adhesive and the resultant plywood met the interior use requirements. However, the tensile shear strength of the plywood bonded by the adhesive with these cross-linkers is about 20% lower than the commercial MUF resin, which has been attributed to the brittleness of the soy protein–based adhesive. In addition, this adhesive brittleness increased when a short chain cross-linker was included and resulted in a low tensile shear strength of the resulting plywood. Therefore, a chemical compound with a long chain structure and multiple active groups may help to increase the toughness of the adhesive, which would improve the water resistance with lower loadings of the additive.

Neopentyl glycol diglycidyl ether (NGDE) is a long-chain epoxide with two epoxy groups at the end of the chain, and it has the advantage of low toxicity and high activity. NGDE is usually used as a reactive diluent of epoxy resin, which brings a high strength and impact resistance to the resulting epoxy resin [23]. In this reported study, NGDE was used as a cross-linker to enhance the soybean meal-based (SM) adhesive. The solids content, viscosity and water resistance of the adhesive were measured. The functional groups, thermo-stability, and fracture surface of the cured adhesive were characterized and analyzed to better understand why the water resistance was improved. Three-layer plywood specimens were fabricated with the resultant adhesives and tested for their bond strength. Furthermore, all the characterizations are compared with the soybean meal/PAE adhesive and commercial MUF resin.

## 2. Materials and Methods

### 2.1. Materials

Soybean meal (SM) was obtained from Xiangchi Grain and Oil Company in Zibo, China, and then milled to a 250 mesh flour. The composition of the soybean meal flour were tested as follows: 46.85% soy protein, 8.86% moisture, 6.46% ash, and 0.56% fat with the remainder were polysaccharide. Neopentyl glycol diglycidyl ether (NGDE), sodium dodecyl sulfate (SDS), and the other chemicals were AR grade reagents and were purchased from Tianjin Chemical Reagent Co. (Tianjin, China). Poplar veneer (40 cm × 40 cm × 1.5 cm, 8% of moisture content) was provided from Wen’an, China.

### 2.2. Preparation of Soybean Meal-Based Adhesive

To prepare the various adhesive samples, soybean meal flour was added to deionized water and stirred for 10 min at 20 °C. Then sodium dodecyl sulfate and NGDE were added sequentially and the mixture was further stirred for 10 min at 20 °C. The adhesive formulations are shown in Table 1.

As a control, a soybean meal–based adhesive (sample 6) with polyamidoamine-epichlorohydrin (PAE) was prepared using the following procedure: soybean meal flour (28 g) was added to the mix of deionized water (22 g) and PAE solution (concentration 12%, 50 g) and stirred for 10 min at 20 °C.

A commercial melamine-urea-formaldehyde (MUF) resin (Sample 7, named ANLIAN-MUF-E1) was used to fabricate E1 level plywood for indoor use. This MUF resin was used to compare with the novel adhesive prepared in this effort. The MUF was obtained from Jiangsu Anlian Wooden Product Co., Ltd. (Xuzhou, China) The synthesis procedure details were described as following: The overall molar ratio of MUF resin was 1.1. In the first step, Formaldehyde (F) was added into a reactor and the pH value was adjusted to 8.0, then the first portion of urea (U1) was added at the molar ratio of 2.0 (F/U). The mixture was heated to 90 °C within 30 min and kept 1 h at a 7.5 pH value. At the second step, the pH value of mixture was adjusted to 3.8 and kept about 1 h to achieve the target viscosity (22 s using Tu-4 cup at 40 °C). At the third stage, the pH value of the mixture was adjusted to 8.0 and the second portion of the urea (U2) with all the melamine (M) was added and further reacted for 40 min. At last, the mixture was cool down to 30 °C and the pH value was adjusted to 7.5. The melamine content was 8 wt % (for the resultant resin). When used to fabricate plywood, 100 g of MUF resin was mixed with 25 g of wheat flour and 1 g of ammonium chloride to produce a mixture for bonding.

### 2.3. Preparation of the Plywood Sample

Three-ply plywood samples were prepared with 120 °C of hot press temperature and 1.0 MPa of hot press pressure [24]. The adhesive spread was different for keeping the same amount of solids adhesive in the veneer (Table 2). The MUF resin was different form the protein based adhesive, so that, the MUF resin (with 25% wheat flour) was 160 g/m^2^ according to the plywood fabrication industry. For the protein based adhesive, the hot pressing time was 90 s per sample thickness (mm) because of the low solids content. For the MUF resin, the hot press time was 60 s per sample thickness (mm), because of its high solids content. After hot pressing, the water content of the resulting plywood was at the range of 8%–10%. The plywood samples were stored under ambient conditions for at least 12 h before testing.

### 2.4. Solids Content Measurement

The adhesive solids content was determined using a weight method from three parallel samples.

### 2.5. Dynamic Viscoelastic Measurement

The apparent viscosity of the various adhesives was determined using a rheometer (Thermo Scientific HAAKE RotoVisco 1, Waltham, MA, USA) with a parallel plate fixture (20 mm diameter). The distance was set to 1 mm for all measurements. Experiments were conducted under a steady shear flow at 25 °C. Shear rates ranged from 1 to 300 s^−1^ in 10 s^−1^ increments. The viscosity value at 1 s^−1^ shear rate was recorded to compare all the adhesives. All of the measurements were conducted in triplicate and the average values were reported.

### 2.6. Water Resistance Measurement

The water resistance of the interior use plywood (Type II plywood) was determined using a tensile shear strength test (WDW-200E, Jinan, China) in accordance with the China National Standards (GB/T 17657-1999). Twelve bonded plywood specimens (2.5 cm × 10 cm) were cut from two plywood panels and submerged into water at 63 ± 2 °C for 3 h and then dried at a room temperature for 10 min before a tension testing. The tensile shear strength was calculated by the following equation:

(1) $$\text{Bonding strength }(\mathrm{MPa})=\frac{\text{Tension Force }(N)}{\text{Gluing area }(m^{2})}$$

### 2.7. Insolubles Measurement

The adhesives were placed in an oven at 120 ± 2 °C until a constant weight was obtained. The cured adhesives were crushed into 10 pieces with a similar size (about 1 g) and weighted (M). These pieces were wrapped with a filter paper and weighted (*n*), then they were soaked in a cup with distilled water for 24 h at ambient temperature. After that the package was taken out and oven-dried at 105 ± 2 °C until a constant weight was obtained (*m*). The residual rate was defined as Equation (2).

(2) $$\text{Insolubles left }(\%)=1-\frac{\text{n}-\text{m}(\text{g})}{\text{M}(\text{g})}\times 100\%$$

### 2.8. Cracks Observation of Cured Adhesive

For visually evaluation of the toughness of cured adhesive, the modified liquid adhesive was uniformly coated on a piece of aluminum foil, and then the coated aluminum foil was placed in an oven at a temperature of 120 ± 2 °C for 2 h. After the adhesive curing, the coated aluminum foil was placed in a desiccator for 30 min to cool down and then photographed using a Digital Single Lens Reflex (Nikon D90, Shanghai, China).

### 2.9. Fourier Transform Infrared (FTIR) Spectroscopy

The adhesive sample was cured in an oven at 120 ± 2 °C until a constant weight was obtained and then ground into a 200 mesh powder. The sample powder mixed with KBr at a mass ratio of 1/70, then pressed in a mold to form a sample folium. The FTIR spectra of the cured adhesive sample was recorded using a Nicolet 7600 spectrometer (Nicolet Instrument Corporation, Madison, WI, USA) from 500 to 4000 cm^−1^ with a 4 cm^−1^ resolution using 32 scans.

### 2.10. Thermogravimetric (TG) Measurement

The adhesive sample was cured in an oven at 120 ± 2 °C until a constant weight was obtained and then ground into a 200 mesh powder. The thermal stability of the cured adhesives was tested using a TGA instrument (TA Q50, WATERS Company, New Castle, DE, USA). About 5 mg of the powdered samples was weighed into a platinum cup and scanned from the room temperature to 600 °C at a heating rate of 10 °C/min in a nitrogen environment while recording the sample weight changes.

### 2.11. Scanning Electron Microscopy (SEM)

The different samples were put on a piece of aluminum foil and cured in an oven at 120 ± 2 °C until a constant weight was achieved. Then the cured adhesive sample was broken into several pieces. A Hitachi S-3400N (Hitachi Science System, Ibaraki, Japan) scanning electron microscope was used to observe fractured surfaces of the adhesive piece. The surface was sputter coated with gold prior to examination under the microscope.

## 3. Results and Discussion

### 3.1. Dynamic Viscoelastic, Solids Content and Insolubles Left Measurement

The viscosity at the 1 s^−1^ shear rate of the adhesives is presented in Table 3. The viscosity of the SM adhesive was recorded at 35,310 mPa·s. After incorporating SDS, the viscosity of the adhesive increased by 293.0% to 138,800 mPa·s. This is due to the SDS partially unfolding the soy protein molecules and exposing active groups inside of the protein molecule. These groups interact with each other and form more intermolecular forces, such as hydrogen bonds and electrostatic interactions, to increase the force between the protein molecules, resulting in a heightened viscosity. In addition, the foaming is observed after adding SDS, which further increased the adhesive viscosity. The viscosity of the adhesive decreased with the increased addition of NGDE. Adding 2 g of NGDE in the adhesive formulation, the viscosity of the adhesive decreased by 85.2% to 20,530 mPa·s compared with the SM/SDS adhesive. When the addition of the NGDE reached 8 g, the viscosity of the adhesive was further reduced by 58.2% to 8583 mPa·s. This indicates that NGDE disperses well in the adhesive. The molecule of NGDE is small when compared with soy protein molecules, resulting in NGDE embedding into the soy protein molecules and reducing intermolecular forces between the protein molecules, thus reducing the frictional resistance within the liquid and presenting a low adhesive viscosity [25,26]. In addition, the NGDE addition reduces the foaming of the adhesive, thus further reducing the viscosity of the resulting adhesive. The viscosity of the SM/PAE adhesive was recorded at 44,840 mPa·s, which was 27.0% higher than that of adhesive 0. The addition of cationic-type PAE increases the intermolecular forces among the protein molecules, which presents a high viscosity of the adhesive. The viscosity of MUF resin was 300 mPa·s, which was the lowest of all adhesives. In this viscosity, MUF resin easily penetrates into the wood surface and thus cannot form an adhesive layer. Therefore, when using MUF resin to bond plywood, approximately a 25 wt % addition of wheat flour is mixed with the MUF resin to increase the adhesive viscosity and prevent over-penetration.

The solids content is an important property of wood adhesive that influences its performance in forming a bond to plywood. The solids contents of the various adhesives are shown in Table 3. The solids content of the SM adhesive was 27.0%. However, the SM adhesive had a high viscosity of 35,310 mPa·s, which caused a flow issue. The solids content of the adhesive increased with the addition of NGDE because adding NGDE was equivalent to increasing the mass of the adhesive. As expected, the solids content of the adhesive increased gradually from 27.0% to 34.2% as the NGDE addition was increased from 2 to 8 g. Moreover, the solids content of the SM/SDS/NGDE-6 adhesive was 33.0%, which was increased by 22.2% over the SM adhesive. The solids content of the SM/PAE adhesive was measured at 33.7%, which was similar to adhesive 4. For the MUF resin, the solids content was recorded at 54.2%, which is the highest one of all the adhesives. MUF resin is water-soluble and has a small molecular weight compared with soy protein, thus presenting a high solids content and low viscosity. The high solids content of MUF resin determines a low hot press time and a better dimensional stability of the resulting plywood.

Water resistance of the adhesive can be measured by the insolubles left of the cured adhesive [27]. Small molecular substances in the cured adhesive are easy to dissolve in water, so that the insolubles left of the adhesive have a high cross-linking structure which determines the water resistance of the adhesive. Table 3 shows the insolubles left of the different cured adhesives. The insolubles left of the SM adhesive was 76.3%. When the denaturing agent SDS was added, the insolubles left of the SM/SDS adhesive increased to 77.5%, which was attributed to the unfolded protein molecules exposing the hydrophobic groups. These hydrophobic groups prevent water intrusion and improve the water resistance of the adhesive. In addition, the unfolded soy protein molecule rearranges during the curing process and forms a larger crystal region [12], which also increases the insolubles left of the adhesive. After adding NGDE, the insolubles left of the SM/SDS/NGDE-2 adhesive increased by 5.4% and increased by another 5.0% when 6 g NGDE was added to the adhesive formulation (SM/SDS/NGDE-6 adhesive). Compared with the SM adhesive, the insolubles left of the SM/SDS/NGDE-6 adhesive was improved by 12.5% and reached 85.8%, which was higher than that of the SM/PAE adhesive and at the same level as the MUF resin. This is attributed to the NGDE possessing epoxy groups that react with the active groups in the protein molecule, forming a more cross-linked structure in the adhesive, which improves the water resistance of the adhesive.

### 3.2. FTIR Spectroscopic Analysis

**Figure 1:**
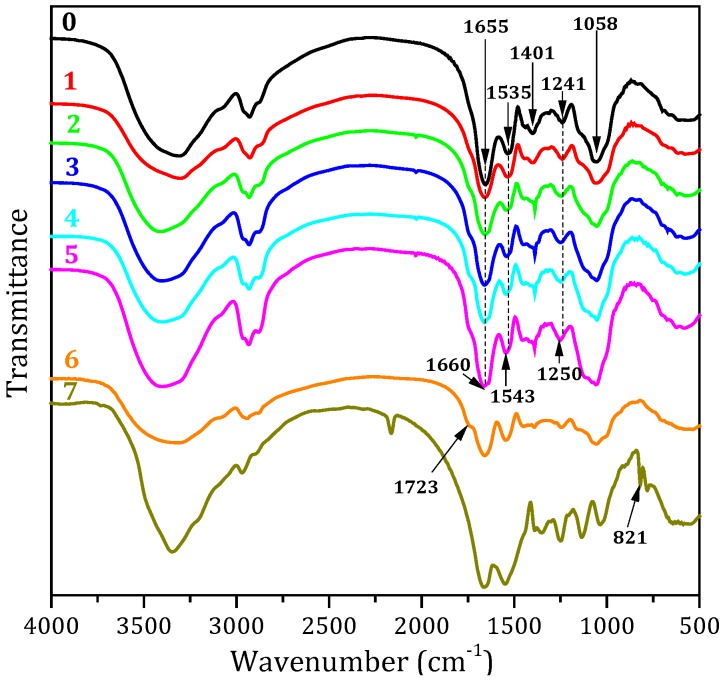
FTIR spectra of the different adhesives: 0 (SM adhesive), 1 (SM/SDS adhesive), 2 (SM/SDS/NGDE-2 adhesive), 3 (SM/SDS/NGDE-4 adhesive), 4 (SM/SDS/NGDE-6 adhesive), 5 (SM/SDS/NGDE-8 adhesive), 6 (SM/PAE adhesive), and 7 (MUF resin).

**Figure 2:**
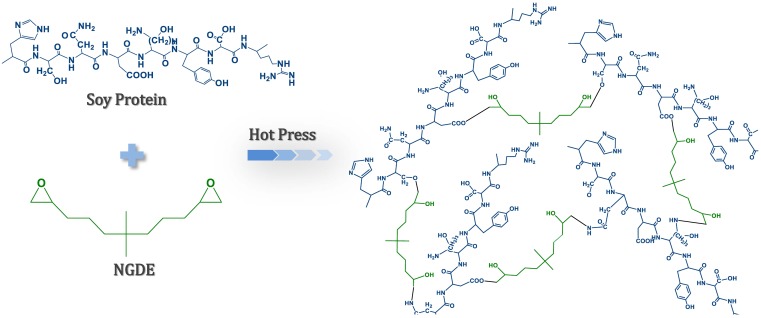
The curing process of the soy-based adhesive enhanced with NGDE.

The FTIR spectra of the different adhesives are presented in Figure 1. The main absorption bands of the peptide were attributed to the peaks approximately at 1656, 1536, and 1241 cm^−1^, which were characteristic of amide I (C=O stretching), amide II (N–H bending) and amide III (C–N and N–H stretching) [28]. The bands corresponding to C–O bending were located at 1059 cm^−1^ [29]. As the NGDE addition was increased from 0 to 8 g in the adhesive, the absorption peak for the C–O bending (1059 cm^−1^) gradually increased, indicating that the NGDE was well distributed in the adhesive system. After adding NGDE, the amide I peak shifted from 1655 to 1660 cm^−1^, while the amide II peak shifted from 1535 to 1543 cm^−1^, and the amide III group shifted from 1241 to 1250 cm^−1^ (blue shift) in the spectrum of the SM/SDS/NGDE-8 adhesive, indicating that there was a much more compact structure formed in the adhesive compared with the SM adhesive [30]. The peak of amide II decreased and the peak of amide III increased in the spectrum of the adhesive with NGDE, indicating that the NGDE reacted with the protein molecule and formed a cross-linked structure (most likely the specific reaction discussed in Figure 2). This cross-linking reaction led to the formation of a denser structure, which is proved by the blue shift of the characterization peak of amide, thus improving the water resistance of the adhesive. Compared with the SM adhesive, no obvious changes were observed at the C–O bending and amide II peak in the spectra of the SM/PAE adhesive; meanwhile, a new minor peak was observed at 1723 cm^−1^, indicating that the reaction process of PAE and NGDE was different. The functional groups of MUF resin were different from the soy protein–based adhesive. The peak at 814 cm^−1^ is the characteristic peak of melamine. During the curing process, the MUF resin molecule forms a cross-linked network by methylene and ether bonds through a self-condensation reaction.

### 3.3. TGA Analysis

**Figure 3:**
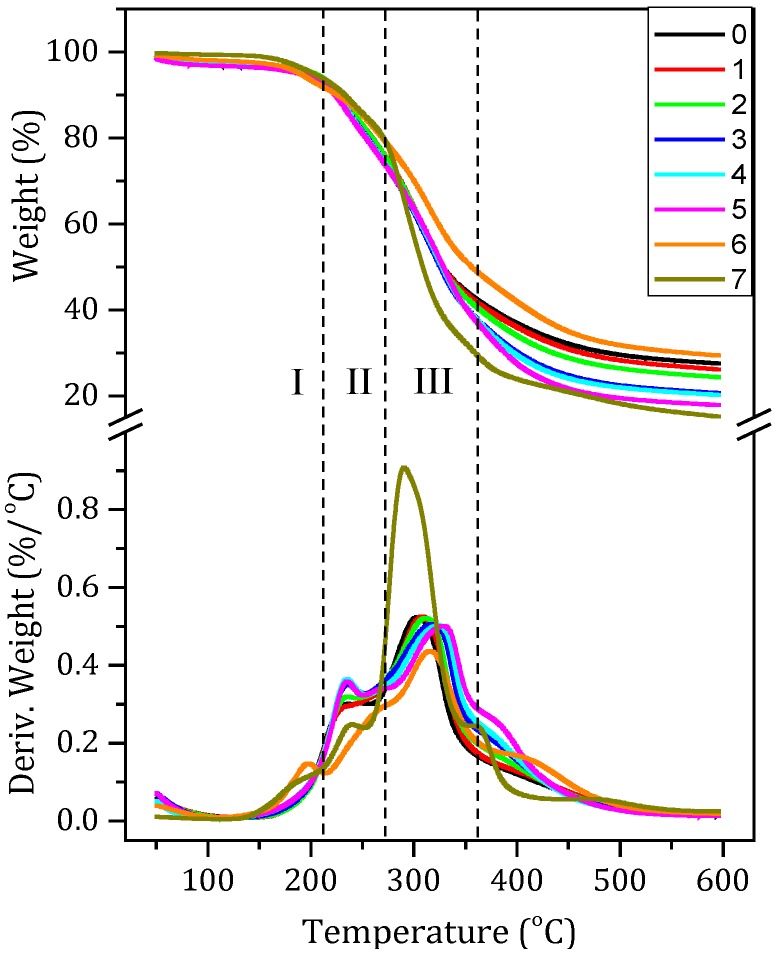
The thermogravimetric (TG) and derivative thermogravimetric (DTG) curves of the different adhesives: 0 (SM adhesive), 1 (SM/SDS adhesive), 2 (SM/SDS/NGDE-2 adhesive), 3 (SM/SDS/NGDE-4 adhesive), 4 (SM/SDS/NGDE-6 adhesive), 5 (SM/SDS/NGDE-8 adhesive), 6 (SM/PAE adhesive), and 7 (MUF resin). I: Possible post-reaction stage, II: Initial degradation stage, III: Skeleton structure degradation stage.

Figure 3 shows the thermogravimetric (TG) and derivative thermogravimetric (DTG) curves of the various adhesives. The thermal degradation process of the adhesives could be divided into three stages. The first stage (I) is a possible post-reaction stage, at a temperature region of 50–210 °C, which is attributed to the possible subsequent thermal reaction of the material. The second stage (II) is an initial degradation stage from 210–270 °C, which results from the weight loss of the degradation of small molecules and the breakage of some unstable chemical bonds. The third stage (III) is the degradation of the skeleton structure of the sample, at a temperature region of 270–360 °C, which results from the thermal degradation of the cross-linked network structure. Before the first degradation stage, a small weight loss occurred, which is attributed to the evaporation of residual moisture. After the third degradation stage, a further heating causes breakages of the C–C, C–N, and C–O linkages and the soy protein backbone peptide bonds decomposed, producing gases such as CO, CO_2_, NH_3_, and H_2_S [31,32].

After adding NGDE in the adhesive formulation (SM/SDS/NGDE-2 adhesive), a strong peak in the second stage was observed compared with the SM adhesive and this peak increased when the NGDE content increased from 2 to 6 g, indicating a new structure formed in the cured adhesive. This new structure is attributed to the chemical reaction between the NGDE and the protein molecule, which improves the water resistance of the adhesive [33]. As the amount of NGDE was further increased from 6 to 8 g, the peak in the second stage showed no obvious difference, indicating that this quantity of NGDE was excessive, leaving residual NGDE in the adhesive. This maybe the reason why the insolubles left of adhesive 5 showed a slight decrease in Figure 1. In the third stage, the thermal degradation behavior showed an evident distinction that was related to the presence of NGDE. When comparing adhesives 0 to 5, the adhesive without NGDE exhibited the highest degradation rate, which decreased significantly when NGDE was incorporated. With the addition of NGDE to the adhesive formulation, the degradation rate decreased and the peak of the third stage moved to a high temperature, suggesting a better thermal stability for the adhesive with NGDE. Compared with the curve of the adhesive with NGDE, a new peak was observed in stage I in the curve of the SM/PAE adhesive, indicating a post-curing process occurred. No peak was observed at stage II and the peak at stage III decreased, suggested that the adhesive with PAE formed a different structure than that with NGDE. The cured MUF resin content ether bond was easy to break and presented a peak at stage II. A big peak was observed at 300 °C, attributed to the breakages of the C–C and C–N in the MUF resin. So, the cured MUF structure is different from the protein-based adhesive.

### 3.4. SEM Analysis

**Figure 4:**
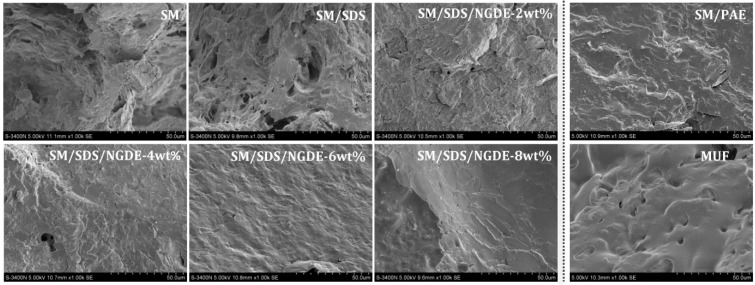
The fracture surface micrograph of the different cured adhesives: 0 (SM adhesive), 1 (SM/SDS adhesive), 2 (SM/SDS/NGDE-2 adhesive), 3 (SM/SDS/NGDE-4 adhesive), 4 (SM/SDS/NGDE-6 adhesive), 5 (SM/SDS/NGDE-8 adhesive), 6 (SM/PAE adhesive), and 7 (MUF resin).

The fracture surface micrographs of the different cured adhesives are shown in Figure 4. A large number of holes and cracks was observed on the fracture surface of the SM adhesive. Moisture could intrude into these holes and cracks, swelling to break the bond, which presented a low water resistance [34]. In the SM adhesive formulation, the protein molecule remains in an intertangling state with a quaternary structure, leading to a non-uniform adhesive system; thus, the whole fracture surface appears very loose and disordered. After introducing NGDE, the fracture surface of the adhesive became compact and no cracks were observed, which effectively prevented moisture intrusion and improved the water resistance of the adhesive. The SM/PAE adhesive showed a similar fracture surface with the adhesive enhanced with NGDE. The cured MUF resin is brittle, so that a more compact and smooth surface is observed in the fracture surface of the MUF resin. Compared with the MUF resin, the wrinkles observed in the fracture surface of the adhesive with NGDE, especially in adhesive 5 and 6, indicated the adhesive with NGDE was flexible.

### 3.5. Crack Observation of Cured Adhesive

**Figure 5:**
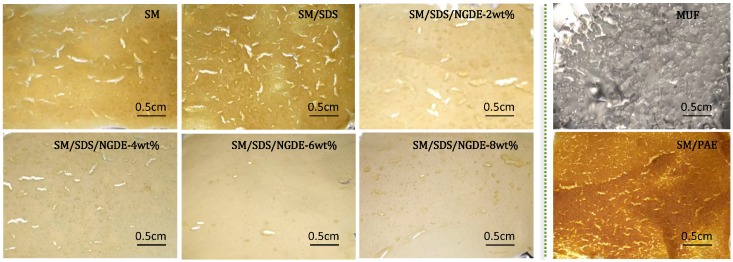
The crack observation of the different cured adhesives: 0 (SM adhesive), 1 (SM/SDS adhesive), 2 (SM/SDS/NGDE-2 adhesive), 3 (SM/SDS/NGDE-4 adhesive), 4 (SM/SDS/NGDE-6 adhesive), 5 (SM/SDS/NGDE-8 adhesive), 6 (SM/PAE adhesive), and 7 (MUF resin).

Figure 5 shows the crack observation of the different cured adhesives. The small cracks were observed in the SM adhesive after curing, indicating that the SM adhesive was brittle. After introducing SDS, the cured adhesive layer showed more cracks, indicating an increase in the brittleness of the adhesive. After adding NGDE, no cracks were observed on the adhesive layer and the surface became more compact and homogeneous, which meant the toughness of the adhesive was improved. A tough adhesive layer improves the impact strength of the adhesive, thus increasing its performance in the bonded plywood. Small cracks were observed in the SM/PAE adhesive layer, indicating the SM/PAE adhesive was brittle when compared with the adhesive with NGDE. The cured MUF resin showed a different surface. The free water and the water produced from the curing process generated bubbles in the adhesive layer, which is presented in Figure 5. The MUF resin formed a smooth layer on the aluminum foil because of its small molecules and uniform distribution. However, when folding a little bit of the aluminum foil, the cured MUF resin layer was easy to break into pieces, indicating the MUF resin was brittle. The same happened at Adhesives 0, 1, 2, 3, 6. For Adhesives 4 and 5, no adhesive pieces fell off and the adhesive layer stuck to the aluminum foil, which proved that Adhesives 4 and 5 were flexible.

### 3.6. Tensile Shear Strength Measurement

**Figure 6:**
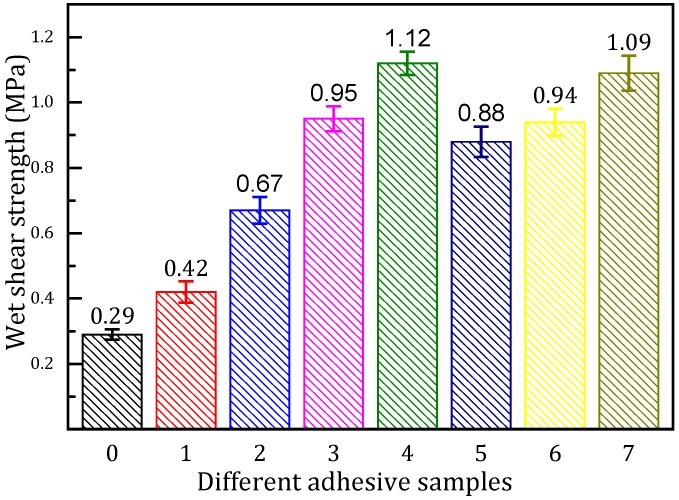
The tensile shear strength of the plywood bonded with the different adhesives: 0 (SM adhesive), 1 (SM/SDS adhesive), 2 (SM/SDS/NGDE-2 adhesive), 3 (SM/SDS/NGDE-4 adhesive), 4 (SM/SDS/NGDE-6 adhesive), 5 (SM/SDS/NGDE-8 adhesive), 6 (SM/PAE adhesive), and 7 (MUF resin).

The tensile shear strength of the plywood bonded with the various adhesives is shown in Figure 6. The tensile shear strength of the plywood bonded with the SM adhesive was 0.29 MPa, which failed to meet the interior use plywood requirement (≥0.7 MPa). After the use of SDS, the tensile shear strength of the plywood was increased by 44.8% to 0.42 MPa, and further increased by 59.5% to 0.67 MPa after 2 g NGDE was added into the adhesive formulation. As the addition of NGDE increased in the adhesive formulation, the tensile shear strength of the resultant plywood increased to 0.95 MPa when 4 g of NGDE was incorporated, which met the interior use plywood requirement. Further increasing the NGDE content resulted in the tensile shear strength of the plywood bonded with the SM/SDS/NGDE-6 adhesive increasing by 286.2% compared to the SM adhesive, and it reached a maximum value of 1.12 MPa. This is due to the NGDE reacting with the active groups in the soy protein molecule to form a denser and more cross-linked network in the adhesive, which improves the water resistance of the adhesive. In addition, using NGDE increased the toughness of the resultant adhesive which resists interior force caused by water intrusion, further increasing the water resistance of the resulting plywood. From another perspective, after adding NGDE, the viscosity of the adhesive is reduced, which helps the adhesive to penetrate into the wood surface and form more interlocks, thus further increasing the tensile shear strength of the resulting plywood [35]. A further increase in the NGDE content to 8 g caused the tensile shear strength of the resultant plywood to decrease to 0.88 MPa, which was attributed to the overly low viscosity leading to the over-penetration in the wood surface. The tensile shear strength of the plywood bonded with 6 g NGDE was 19.1% higher than that of the 6 g PAE, which proved that a chemical compound with multiple epoxy groups and a long chain structure offers a better water resistance for the resulting plywood. The tensile shear strength of the plywood bonded by the soybean meal-based adhesive with 6 g NGDE was at the same level as the commercial MUF resin, indicating that the soybean meal–based adhesive could be used as a plywood adhesive.

## 4. Conclusions

NGDE as a cross-linker effectively increases the water resistance of a soybean meal-based adhesive, which is attributed to the formation of a dense cross-linking network by the reaction between the epoxy group of NGDE and the active groups on soy protein molecules. The addition of NGDE creates a smooth and denser fracture surface in the cured adhesive, thus further increasing the water resistance of the adhesive. The 6 g NGDE addition improved the water resistance of the adhesive by 12.5%. In addition, when using the resulting adhesive to bond plywood, the tensile shear strength was increased by 286.2% to 1.12 MPa, which met the interior use plywood requirement. This enhancement is due to the improvement of the adhesive water resistance and toughness, and the reduction of viscosity. When compared to the other adhesives, the tensile shear strength of the plywood bonded using the soybean meal-based adhesive with 6 g NGDE was 19.1% higher than that with 6 g PAE, and was similar to the commercial MUF resin.

## Figures and Tables

**Table 1:** Various adhesive formulations.

Sample		Adhesive Formulation				
		Soybean meal flour (g)	Deionized water (g)	Sodium dodecyl sulfate (g)	NGDE (g)	PAE solution (g)
0	SM adhesive	28	72	–	–	–
1	SM/SDS adhesive	28	72	1	–	–
2	SM/SDS/NGDE-2 adhesive	28	72	1	2	–
3	SM/SDS/NGDE-4 adhesive	28	72	1	4	–
4	SM/SDS/NGDE-6 adhesive	28	72	1	6	–
5	SM/SDS/NGDE-8 adhesive	28	72	1	8	–
6	SM/PAE adhesive	28	22	–	–	50
7	MUF resin	Molar ratio of F/(U+M):1.1; M additon is 8% for the resultant resin				

SM: Soybean meal; SDS: Sodium dodecyl sulfate; NGDE: Neopentyl glycol diglycidyl ether; PAE: polyamidoamine-epichlorohydrin; MUF resin: Melamine urea formaldehyde resin; F/(U+M): Formaldehyde/(urea+melamine).

**Table 2:** The adhesive spread of the different adhesives, 0–7.

Adhesive Sample	0	1	2	3	4	5	6	7
Adheisve spread (g/m^2^)	260	249	234	220	212	205	208	160

**Table 3:** The viscosity at 1 s^−1^ shear rate, the solids content and insolubles left of the different adhesives: 0 (SM adhesive), 1 (SM/SDS adhesive), 2 (SM/SDS/NGDE-2 adhesive), 3 (SM/SDS/NGDE-4 adhesive), 4 (SM/SDS/NGDE-6 adhesive), and 5 (SM/SDS/NGDE-8 adhesive), 6 (SM/PAE adhesive), 7 (MUF resin).

Adhesive Sample	0	1	2	3	4	5	6	7
Initial viscosity (mPa·s)	35,310	138,800	20,530	19,230	13,090	8583	44,840	300
Solids content (%)	27.0	28.1	29.9	31.7	33.0	34.2	33.7	54.2
Insolubles left (%)	76.3	77.5	81.7	84.3	85.8	84.7	84.4	86.1
